# Pethidine dose and female sex as risk factors for nausea after esophagogastroduodenoscopy

**DOI:** 10.3164/jcbn.18-5

**Published:** 2018-06-20

**Authors:** Toshihiro Nishizawa, Hidekazu Suzuki, Masahide Arita, Yosuke Kataoka, Kazushi Fukagawa, Daisuke Ohki, Keisuke Hata, Toshio Uraoka, Takanori Kanai, Naohisa Yahagi, Osamu Toyoshima

**Affiliations:** 1 Gastroenterology, Toyoshima Endoscopy Clinic, 6-17-5 Seijo, Setagaya-ku, Tokyo 157-0066, Japan; 2 Department of Gastroenterology, National Hospital Organization Tokyo Medical Center, 2-5-1 Higashigaoka, Meguro-ku, Tokyo 152-8902, Japan; 3 Division of Research and Development for Minimally Invasive Treatment, Cancer Center, Keio University School of Medicine, 35 Shinanomachi, Shinjuku-ku, Tokyo 160-8582, Japan; 4 Division of Gastroenterology and Hepatology, Department of Internal Medicine, Keio University School of Medicine, 35 Shinanomachi, Shinjuku-ku, Tokyo 160-8582, Japan; 5 Medical Education Center, Keio University School of Medicine, 35 Shinanomachi, Shinjuku-ku, Tokyo 160-8582, Japan; 6 Department of Gastroenterology, Graduate School of Medicine, The University of Tokyo, 7-3-1 Hongo, Bunkyo-ku, Tokyo 113-0033, Japan; 7 Department of Surgical Oncology, Graduate School of Medicine, The University of Tokyo, 7-3-1 Hongo, Bunkyo-ku, Tokyo 113-0033, Japan

**Keywords:** pethidine, nausea, endoscopy

## Abstract

Nausea and vomiting after esophagogastroduodenoscopy have not been studied in detail. The aim of this study was to evaluate the risk factors for post-endoscopic nausea. We performed a case-control study at the Toyoshima Endoscopy Clinic. Eighteen patients with post-endoscopic nausea and 190 controls without post-endoscopic nausea were analyzed. We conducted univariate and multivariate logistic regression analyses with respect to patient age; sex; body height; body weight; the use of psychotropic drugs as baseline medications; and the dosing amounts of midazolam, pethidine, flumazenil and naloxone. On univariate analysis, post-endoscopic nausea was significantly related with patient age (odds ratio = 0.946); female sex (odds ratio = 10.85); body weight (odds ratio = 0.975); and the dose per kg body weight of pethidine (odds ratio = 53.03), naloxone (odds ratio = 1.676), and flumazenil (odds ratio = 1.26). On multivariate analysis, the dose per kg body weight of pethidine (odds ratio = 21.67, *p* = 0.004) and female sex (odds ratio = 13.12, *p* = 0.047) were the factors independently associated with post-endoscopic nausea. The prevalence of nausea after esophagogastroduodenoscopy was 0.49% (18/3,654). In conclusion, post-endoscopic nausea was associated with the dose of pethidine and female sex.

## Introduction

Esophagogastroduodenoscopy (EGD) is an important medical tool in the screening, diagnosis, and treatment of a variety of gastrointestinal diseases.^(1,2)^ Reported post-endoscopic complications include throat pain, nausea, and headache. Though some these are rare, the rate of post-endoscopic nausea, which is considered to be relatively major among the different post-endoscopic complications, has been reported to be 1.5%.^(3)^ Post-endoscopic nausea could be caused by the use of a peri-endoscopic sedative and analgesic medications, air insufflation, and pharyngeal stimulation.^(4)^ Post-endoscopic nausea is one of the most undesirable complications, and can complicate management after EGD, delaying discharge and recovery. Furthermore, post-endoscopic nausea can lead to refusal to undergo repeat EGD. In rare cases, severe nausea after EGD may require hospitalization. Following surgery with anesthesia, post-operative nausea and vomiting (PONV) affects about 20–40% of patients. Extensive literature about PONV suggests prophylactic strategies and pharmacological management tailored to the patient’s risk level.^(5)^ However, no reported study has examined post-endoscopic nausea. Thus, the aim of this study was to evaluate the risk factors associated with post-endoscopic nausea.

## Methods

### Subjects

We performed a case-control study at the Toyoshima Endoscopy Clinic. Between May 2016 and April 2017, 3,654 patients underwent EGD. Among them, patients with post-endoscopic nausea were enrolled in the current study. The control group included consecutive patients who underwent EGD between April 11, 2017 and April 28, 2017. The diagnostic criteria for post-endoscopic nausea include grade 2–3 nausea or vomiting within 12 h after EGD. Grade 1 nausea is defined as loss of appetite without alteration in eating habits according to common terminology criteria for adverse events (CTCAE). Grade 2 nausea is defined as decreased oral intake without dehydration. Grade 3 nausea is defined as inadequate oral intake with an indication for tube feedings or hospitalization. Grade 4 is defined as life-threatening consequences. Patients who simultaneously underwent EGD and colonoscopy were excluded.

The following demographic and clinical characteristics were collected from medical records: patient age; sex; body height; body weight; body mass index (BMI); the use of psychotropic drugs as baseline medications; and the administered doses of midazolam, pethidine, flumazenil and naloxone. Written, informed consent was obtained from each patient included in the study. This study was approved by Ethical Review Committee of Hattori Clinic (September 7, 2017), and conformed to the ethical guidelines of the 1975 Declaration of Helsinki as reflected in a prior approval by the institution’s human research committee.^(6)^

### Endoscopic examination

EGD was performed by 14 experienced endoscopists. EGD was performed as a screening method during a health evaluation, for follow-up of gastritis and/or gastric tumor, for the examination for abdominal symptoms, to investigate an abnormality of photoﬂuorography, to examine abnormal serum pepsinogen levels, or due to a positive finding of *H. pylori* antibody. The pharynx of the patients was topically anesthetized with a gargle of lidocaine hydrochloride 2% viscous solution (Xylocaine^®^ Viscous 2%; AstraZeneca Inc., Cambridge, UK) before the EGD.^(7)^ The endoscopists were allowed to use their clinical judgement to decide the amount and type of sedative and analgesic medication and the antagonist—midazolam (0–10 mg), pethidine (0–70 mg), flumazenil (0–0.5 mg) and naloxone (0–0.4 mg)—to be used. Following the EGD, the patients were transferred to the recovery room. All adverse events including nausea and vomiting were evaluated by the recovery room nurse. Patients were requested to return 10 to 14 days later for the explanation of their EGD results and were also interviewed regarding any additional adverse events.

### Statistical analysis

We evaluated the effects of patient age; sex; body weight; BMI; the use of psychotropic drugs as baseline medications; and the dose per kg body weight of midazolam, pethidine, flumazenil, and naloxone on post-endoscopic nausea. The clinical parameters were analyzed via the chi-square or univariate logistic regression analysis. The predictors found to be associated with post-endoscopic nausea on univariate analysis (*p*<0.1) were subsequently assessed using a multiple logistic regression method to identify independent factors.^(8)^ Patient age, body weight, BMI, and the dose per kg body weight of each drug were included as continuous variables in the univariate and multivariate logistic regression analyses. A *p* value of less than 0.05 was considered statistically significant. The data were analyzed using the StatMate IV software (ATOMS, Tokyo, Japan).

## Results

Of the 25 eligible patients, seven were excluded because they simultaneously underwent EGD and colonoscopy. Of the 274 controls, 84 were excluded because they simultaneously underwent EGD and colonoscopy. Finally, 18 patients with post-endoscopic nausea and 190 controls without post-endoscopic nausea were analyzed.

Table 1 shows the univariate and multivariate analysis results for post-endoscopic nausea. On univariate analysis, post-endoscopic nausea was significantly related with patient age (odds ratio = 0.946, *p* = 0.0054), female sex (odds ratio = 10.85, *p* = 0.022), body weight (odds ratio = 0.975, *p* = 0.0511), the dose per kg body weight of pethidine (odds ratio = 53.03, *p*<0.001), the dose per kg body weight of naloxone (odds ratio = 1.676, *p*<0.001), and the dose per kg body weight of flumazenil (odds ratio = 1.26, *p* = 0.0374).

On multivariate analysis, the dose per kg body weight of pethidine [odds ratio = 21.67, 95% confidence interval (CI) = 2.547–184.3, *p* = 0.005] and female sex (odds ratio = 13.12, 95% CI = 1.035–166.2, *p* = 0.047) were independently associated with post-endoscopic nausea (Table 2).

The prevalence of nausea after EGD was 0.49% (18/3,654) in this study. Two patients received only pethidine, 1,735 patients received both pethidine and midazolam, 1,316 patients received only midazolam, and 601 patients received neither pethidine nor midazolam. Among patients who did not receive pethidine, the prevalence was 0.21% (4/1,917). Among those receiving pethidine, the prevalence was 0.81% (14/1,737). The prevalence in patients receiving pethidine was significantly higher than that in patients who did not receive pethidine (*p* = 0.019).

## Discussion

This is a first report about post-endoscopic nausea. In this study, the dose per kg body weight of pethidine and female sex were found to be independent risk factors for the onset of post-endoscopic nausea.

Peri-endoscopic sedative and analgesic medications have often been used to provide patient comfort, reduce procedure time, and improve examination quality during EGD.^(9–11)^ Benzodiazepines such as midazolam are the most commonly used sedatives,^(12,13)^ and these are generally given to the patient along with an opiate (pethidine or fentanyl) for synergism.^(14,15)^ Two randomized controlled trials compared sedation with midazolam plus pethidine versus midazolam alone.^(16,17)^ Sedation with midazolam and pethidine led to significantly less retching, which interfered with the procedure, and endoscopists reported favoring the use of both medications over the use of midazolam alone. However, adverse effects of opiates include nausea and vomiting. Opiates mainly inhibit the neurotransmission of pain by binding to specific opioid receptors that are present in the central nervous system and peripheral tissues.^(18)^ Nausea and vomiting resulting from stimulation of the medullary chemoreceptor trigger zone occur in a dose-independent manner.^(19)^ We also found that post-endoscopic nausea was associated with the dose of pethidine.

In this study, women experienced post-endoscopic nausea more often than men. Silva *et al.* reported that the risks of postoperative nausea and vomiting were associated with female sex in surgery and general anesthesia settings.^(20)^ The observed sex differences could be explained by the presence of a different socialization process for men and women that influences the willingness to communicate distress.^(21)^ Women report more pain than men,^(22)^ and describe more numerous somatic symptoms than men.^(21)^ Other possible explanations include the interaction between sex hormones and opiates and the hormone fluctuations associated with the menstrual cycle.^(23)^

The limitations of this study include its retrospective and case-control design. A follow-up study should be performed prospectively to confirm and clarify the characteristics of nausea and vomiting after EGD.

In conclusion, we found that post-endoscopic nausea was associated with the dose of pethidine and female sex. Endoscopists should recognize that the use of high-dose opiates in female patients might provoke nausea and vomiting after EGD.

## Figures and Tables

**Table 1:** Univariate analysis on post-endoscopic nausea

Variables	Case	Control	Univariate analysis		
			Odds ratio	95%CI	*p* value
Age	45.5 ± 9.2	56.3 ± 15.3	0.946	0.909–0.984	0.0054
Sex (female)	17 (94.4%)	116 (61.1%)	10.85	1.413–83.22	0.022
Body weight	51.4 ± 7.1	56.7 ± 11.1	0.975	0.893–1.000	0.0511
Body mass index	20.3 ± 3.0	21.6 ± 3.2	0.861	0.717–1.033	0.106
Use of psychotropic drugs	1 (5.6%)	36 (18.9%)	0.252	0.032–1.953	0.187
Pethidine (mg/kg)	0.548 ± 0.268	0.193 ± 0.253	53.03	8.973–313.4	<0.001
Naloxone(µg/kg)	3.51 ± 2.5	1.13 ± 1.81	1.676	1.298–2.165	<0.001
Midazolam (mg/kg)	0.057 ± 0.017	0.053 ± 0.048	3.63	0.0009–14974	0.759
Flumazenil (µg/kg)	2.65 ± 3.54	1.69 ± 1.74	1.26	1.014–1.567	0.0374

**Table 2:** Multivariate analysis on post-endoscopic nausea

Variables	Multivariate analysis		
	Odds ratio	95%CI	*p* value
Age	0.963	0.920–1.009	0.114
Sex (female)	13.12	1.035–166.2	0.047
Body weight	1.069	0.9829–1.162	0.12
Pethidine (mg/kg)	21.67	2.547–184.3	0.005
Naloxone (µg/kg)	1.231	0.891–1.702	0.208
Flumazenil (µg/kg)	1.101	0.845–1.435	0.476
